# Aerococcus Urinae Infection Causing Malodorous Urine in a Child: A Case Report

**DOI:** 10.7759/cureus.55635

**Published:** 2024-03-06

**Authors:** Rajesh Dudani, Amani Qasem, Kingsley Udom

**Affiliations:** 1 Department of Pediatrics, John H. Stroger, Jr. Hospital of Cook County, Chicago, USA

**Keywords:** pathogen, urogenital disorders, pediatric, malodorous urine, aerococcus urinae infection

## Abstract

*Aerococcus urinae* (*A. urinae*) infection, primarily observed in elderly patients, is a rare yet emerging occurrence in the pediatric population. Advances in laboratory techniques have facilitated the increased identification of these bacteria in human infections. There have been only a few recent cases reported among children. The scarcity of literature on the clinical presentation and management of such infections in children presents a challenge for pediatricians. Here, we present the case of a 15-month-old male child with Down syndrome who presented with malodorous urine but lacked other typical symptoms of urinary tract infection. Upon investigation, urine analysis revealed pyuria, and urine culture confirmed *A. urinae* infection. The patient also exhibited underlying bilateral mild to moderate hydronephrosis. Successful treatment was achieved with a three-day course of amoxicillin, leading to symptom resolution. This case underscores the significance of promptly identifying *A. urinae* infection in pediatric patients presenting with malodorous urine, as a timely intervention with a short course of treatment may avert more severe and invasive infections.

## Introduction

*Aerococcus urinae* (*A. urinae*) infection is a rare occurrence among pediatric patients [1]. Males are predominately affected in this age group [1]. The most common clinical manifestations in pediatric cases include asymptomatic bacteriuria and malodorous urine, although more severe presentations such as urinary tract infections and endocarditis can occur [1-3]. Currently, there are no established guidelines for optimal management strategies regarding the choice of antibiotics and duration of treatment in pediatric patients. Recurrence is a concern, especially among patients with underlying urogenital disorders [1]. Overall, *A. urinae* is considered an unusual low-grade pathogen in the pediatric population, but it can lead to serious invasive infections.

## Case presentation

A 15-month-old African American male with Down syndrome and a history of ventricular septal defect repair was admitted due to failure to thrive. The child had a history of constipation for two months, which improved after the mother started giving prune juice a week ago. He also exhibited malodorous urine for the past few weeks without an increase in frequency or dysuria. There was no reported history of fever, chills, vomiting, or decreased appetite. The child was born prematurely at 32 weeks' gestation and was exclusively formula-fed until 13 months of age with limited nutritional intake. Additionally, the child had motor and speech delay.

Upon initial examination, the child's vital signs were unremarkable, but his weight was below the 10th percentile. The child exhibited malnourishment with thin extremities and mild abdominal distension. Other notable findings included a reducible umbilical hernia, mild hepatomegaly, a mid-sternal scar consistent with prior cardiac surgery, and bilateral undescended testes.

**Figure 1:**
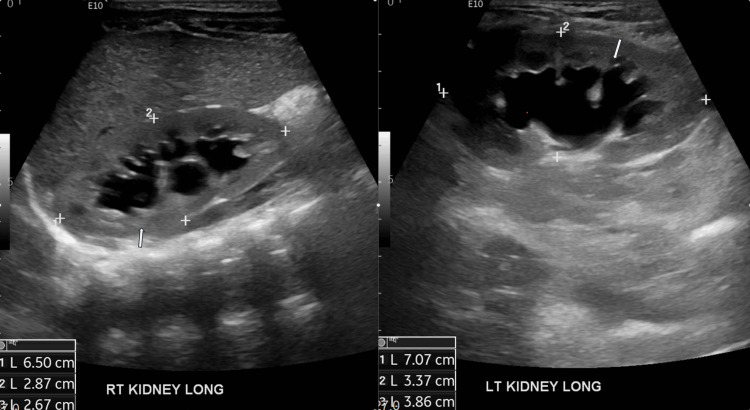
Renal ultrasound showing bilateral mild to moderate hydronephrosis.

**Figure 2:**
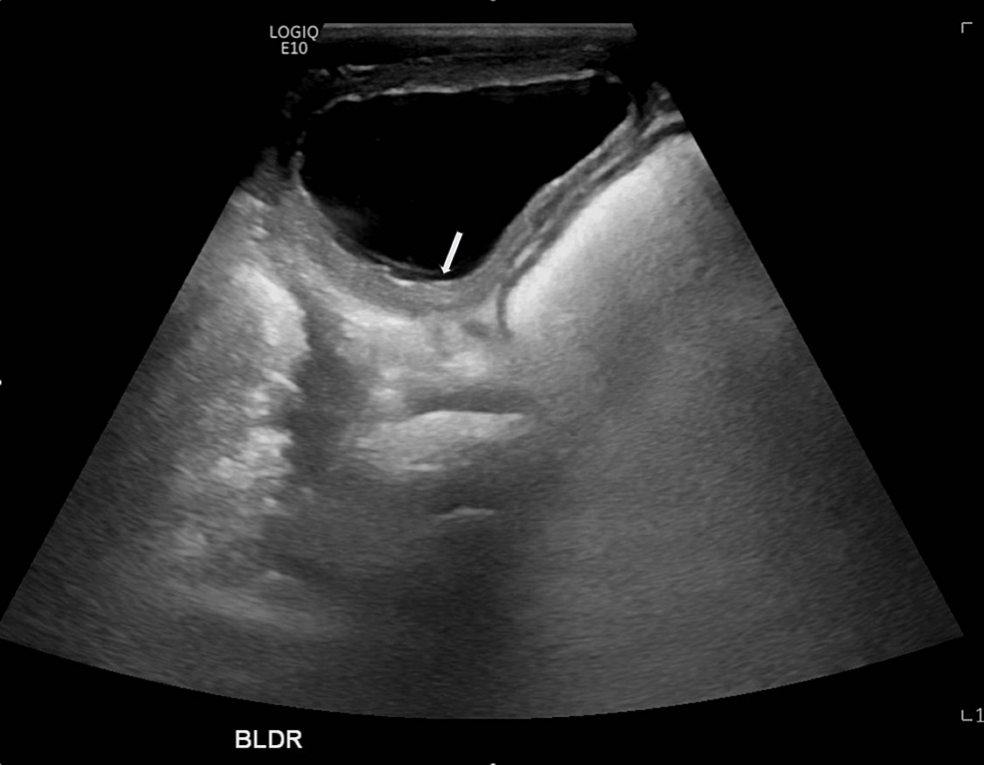
Ultrasound showing thickened bladder wall.

Initial investigations, including complete blood count and complete metabolic panel, were unremarkable. Urine analysis revealed positivity for nitrite and leukocyte esterase, with mild pyuria (WBC 15), and urine culture confirmed the presence of *A. urinae* bacteria. Renal ultrasound indicated bilateral mild to moderate hydronephrosis (Figure 1) and a thickened bladder wall (Figure 2), while a voiding cystourethrogram showed no abnormalities. The patient was treated with amoxicillin for three days, considering cystitis, resulting in the resolution of malodorous urine. Subsequent outpatient follow-up confirmed negative urine culture after two weeks.

## Discussion

*A. urinae* is a Gram-positive, alpha-hemolytic, and catalase-negative bacterium primarily reported in elderly patients [4]. Morphotypic similarities with streptococci, enterococci, and coagulase-negative staphylococci have likely led to the underestimation of this bacterial infection. Several cases reported in the literature initially misidentified these bacteria as others, primarily streptococci [5]. The introduction of matrix-assisted laser desorption ionization time-of-flight mass spectrometry (MALDI-TOF MS) has increased the identification of these bacteria in human infections [5]. While traditionally considered a low-grade pathogen, it has been associated with urinary tract infections and other invasive infections [1-3].

*A. urinae* infections in pediatric patients are infrequent, with scant literature available on clinical presentation and management in this population [1-3,6-10]. While reports in elderly patients encompass both males and females [4], our case involved a male child, aligning with the majority of pediatric cases documented [1]. The underlying cause of this gender variance between geriatric and pediatric cohorts remains elusive. In the pediatric population, it has been associated with asymptomatic bacteriuria, malodorous urine, pyelonephritis, septicemia, and endocarditis [1-3,6-10]. In our case, malodorous urine and failure to thrive prompted consideration of a metabolic disorder. Timely identification of *A. urinae* in urine culture and proper history of nutritional intake prevented extensive investigations. The association of urogenital disorders with *A. urinae* detection in urine is widely reported [1], as seen in our patient who had mild to moderate hydronephrosis (Figure 1). Although evidence regarding antibiotic choice and duration of treatment in pediatric patients is limited [1-3,6-10], our patient responded well to a three-day course of amoxicillin, with clinical improvement and negative urine culture after two weeks. In the majority of invasive aerococcal infections, a urinary tract focus is suspected. Treating cases with malodorous urine may be crucial to prevent serious invasive infections and alleviate psychological strain. Clinical monitoring is essential due to the risk of recurrence, particularly in patients with underlying urogenital disorders [1]. At a three-year follow-up well-child visit, our patient remained asymptomatic.

## Conclusions

This case emphasizes the significance of promptly identifying *A. urinae* infection in pediatric patients presenting with malodorous urine, as a timely intervention with a short course of treatment may prevent more serious infections. Currently, there are no established guidelines and further research is warranted to establish optimal management strategies in pediatric *A. urinae* infections.
